# Gluten Foods

**Published:** 1892-11

**Authors:** Ephraim Cutter

**Affiliations:** President of the American Branch of the Society of Science, Letters, and Art, of London; Corresponding Member of the Belgian and Italian Microscopical Societies, etc., etc., New York


### SELECTIONS.

### Gluten Foods.

By Ephraim Cutter, M.D., LL.D.,
President of the American Branch of the Society of Science, Letters, and Art, of London; Corresponding Member of the Belgian and Italian Microscopical Societies, etc., etc., New York.

### WHAT IS GLUTEN?

The chemist says it is "the viscid, tenacious substance which gives adhesiveness to dough. It may be separated from the flour of grain by subjecting this to a current of water, the starch and other soluble matters being washed out. Gluten consists of glutin—a white substance resembling albumen, precipitated from an alcoholic solution of gluten by the addition of water—vegetable fibrin, and casein, with sometimes a fatty substance."

The morphologist (*morphus*, form; *logos*, account) studies the form elements of substances by the microscope.

Ordinary inspection is morphological, and differs from chemical as follows: Ask a chemist to analyze the contents of a court room. He would take the judge, jury, officers, plaintiff and defendant, the furniture, fixtures, floor, etc., reduce them to ashes by burning, test with reagents, balances, etc., and tell how much iron, potash, sulphur, and other chemical elements there are and their proportions. But the morphologist would look in the court room and say, There is Judge A; Mr. B, foreman of the jury; Mr. C, counsel for, and Mr. D, counsel against; there is Mr. E, sheriff; Mr. F, policeman; there are Messrs. G, H, and I among the spectators; there is a table; there are books, cushions, gas fixtures, plasterings, windows, walls, and so on. The morphologist would analyze the court room immediately by sight and distinguish its contents, while the chemist would present no individualities or distinctions by common names of forms such as everybody knows. Both chemical and morphological analyses complement each other. They do not antagonize. They should in all cases be taken together. The morphological examination of things is the common every-day inspection made in becoming acquainted with life.

The word "flour" means wheat flour.

Start the morphologist on flour and he will tell you that gluten is found in two forms:

1. In cells, called gluten cells, which surround the substance or parenchyma of the grain and contain the starch grains of various sizes mixed in with.

2. The second form of gluten, *i.e.*, granular and free minute dark-colored grains hard to distinguish from the same-sized starch grains. Iodine stains gluten granules yellow and starch grains blue.

The contents of the gluten cell ($\frac{1}{675}$ inch in diameter) are made up of granular gluten $\frac{1}{15000}$ inch in diameter on an average.

In the wheat envelope some make six coats, *i.e.*:

1. Epicarp, or outer coat.
2. Mesocarp, or inner coat of longitudinal cells.
3. Endocarp, transverse cells; cigar coat.
4. Episperm, testa; color coat.
5. Tegmen, gluten; comb coat.
6. Gluten cells, perisperm.

Now, it is evident that coats 1, 2, 3, and 4 should not be found in pure gluten flour, nor should starch, 4 and 5. Granular gluten and in cells should be all the form elements found in *pure* gluten flour.

There are, however, mechanical difficulties which are in the way of completely separating starch and 1, 2, 3, and 4 from the gluten. One of these is the deep longitudinal groove in the wheat which bulges at the bottom and comes close together at the top. When the wheat is soaked and rubbed between the folds of rough cloth, 1 and 2 outer coats are readily removed with the beard. But the groove cannot be cleared. Coat 3 can be removed, with the exception of the groove, and with it goes the germ or plumule and radicle of the embryo.

So in pure gluten flour one must expect to find 5, or gluten coat, and 6, gluten cells. There should also be present the parenchymatous gluten, which is present in all flour, and without which bread could not be made. Starch entirely separate from gluten will not make bread, as it has no viscosity, will not vesiculate or form into bubbles from the retention of air, carbonic acid gas, steam or vaporized alcohol, all of which, forming in, make the dough rise by distending the sponge of gluten filled with starch.

Now, this gluten is obtained, as stated in our definition, by washing out the starch from the dough by a stream of water. It is rich in phosphates, soda, potash, and other mineral ingredients which go to make up healthy normal tissues and which are needful to make a fully equipped human body. This leads us to ask :

### WHAT IS THE VALUE OF GLUTEN?

Magendie, the immortal French physiologist, says gluten by itself secures complete and prolonged nutrition.

Pereira, the great authority on materia medica in his time, says gluten is easy of digestion, and substances which contain it largely are readily digested even by invalids and dyspeptics.

Gluten is rich in phosphates and the mineral salts in a soluble form, which, as said before, are needed to maintain health. It is soluble mineral food. For plants it is a great thing to get soluble mineral food as farmers well know. So is it for man. Again, nitrogen exists in gluten. Although nitrogen is largely taken into the system by means of the lungs, it is not wholly settled as to the amount, and therefore we must rely on what we eat and drink for the nitrogen which is found so abundantly in the body tissues. Hence the value of gluten in the food.

Starch alone is incapable of sustaining life, but combined with gluten, as found in whole wheat, it sustains life indefinitely.

### THE LAW OF THE BEAUTIFUL IN FOOD.

It is a curious ethical fact that the eye has set standards of food. It was so with Mother Eve in the Garden of Paradise. It is so with the fair daughters of Eve who bless the world and govern the diet in homes. Domestic ethics has ordained that flour bread must be white and light. But gluten in normal quantities makes the bread dark. This color does not answer the ethical requirements of modern society, hence starch takes precedence of gluten as a food, and people choose to live on food impoverished to a great extent of its gluten, regardless of the ills that come from trying to live on a food which does not possess the nutritive salts and organic compounds above named in normal quantities to balance the starch.[1]

In the writer's opinion the ills of defective eyes, teeth, hair, bones, cartilages, etc., or, in other words, the defects that are charged to modern civilization, come more from the withdrawal of gluten, from flour, which is such a general article of food. Not all the gluten is withdrawn from flour, else dough could not be made; but too much is withdrawn for health-giving purposes. The mills of fashion in food grind so fine, and fashions in food have such ruts, that to get people generally to give up the idea of whiteness of their flour-bread is as impossible as it is to get ladies to give up corsets. Hence any efforts to restore the lost gluten to the food should be warmly welcomed by all physicians.

There are many such preparations in the market, with so many claims to peerless superiority over each other that the mind is bewildered to know what is best. It is noticed that most of the so-called gluten foods do not come up to their own standards as stated on their labels. This is so true that the writer hitherto has morphologically found whole wheat ground fine, with all the coats left in after going through the smutting mills, the best gluten food. The Arlington wheat meal is a fair type of these meals. The writer, after careful studies of the morphologies of fæces of people who have subsisted on wheat meals, is not prepared to join in the wholesale denunciation of the presence of the coats of wheat in the bread. So far as their mechanically irritative effects are concerned, there is plenty of mucus to envelop such irritating substances, which are not so irritating as the intes-

---

1 See "Is Flour our Proper Food?" Transactions New Hampshire State Medical Society, 1875; The Doctor, New York, December, January, and February, 1889-1890.

tinal calculi shown in the museum of the Royal College of Physicians, Lincoln's Inn Fields, London. Still, meals do not make as good bread as white wheat flours, unless in very skilful hands. Moreover, so long as the curious ethics of modern times places the difficult art of breadmaking in the hands of the lowest class of unskilled labor—the ordinary careless, time serving, unthinking queens of the kitchen—it becomes an important question.

